# Genomic and transcriptomic survey of an endophytic fungus *Calcarisporium arbuscula* NRRL 3705 and potential overview of its secondary metabolites

**DOI:** 10.1186/s12864-020-06813-6

**Published:** 2020-06-24

**Authors:** Jin-Tao Cheng, Fei Cao, Xin-Ai Chen, Yong-Quan Li, Xu-Ming Mao

**Affiliations:** 1 Institute of Pharmaceutical Biotechnology, School of Medicine, Zhejiang University, Hangzhou, 310058 China; 2 Zhejiang Provincial Key Laboratory for Microbial Biochemistry and Metabolic Engineering, Hangzhou, 310058 China

**Keywords:** Endophytic fungus, *Calcarisporium arbuscula*, Genome, Transcriptome, Secondary metabolite

## Abstract

**Background:**

Secondary metabolites as natural products from endophytic fungi are important sources of pharmaceuticals. However, there is currently little understanding of endophytic fungi at the omics levels about their potential in secondary metabolites. *Calcarisporium arbuscula*, an endophytic fungus from the fruit bodies of Russulaceae, produces a variety of secondary metabolites with anti-cancer, anti-nematode and antibiotic activities. A comprehensive survey of the genome and transcriptome of this endophytic fungus will help to understand its capacity to biosynthesize secondary metabolites and will lay the foundation for the development of this precious resource.

**Results:**

In this study, we reported the high-quality genome sequence of *C. arbuscula* NRRL 3705 based on Single Molecule Real-Time sequencing technology. The genome of this fungus is over 45 Mb in size, larger than other typical filamentous fungi, and comprises 10,001 predicted genes, encoding at least 762 secretory-proteins, 386 carbohydrate-active enzymes and 177 P450 enzymes. 398 virulence factors and 228 genes related to pathogen-host interactions were also predicted in this fungus. Moreover*,* 65 secondary metabolite biosynthetic gene clusters were revealed, including the gene cluster for the mycotoxin aurovertins. In addition, several gene clusters were predicted to produce mycotoxins, including aflatoxin, alternariol, destruxin, citrinin and isoflavipucine. Notably, two independent gene clusters were shown that are potentially involved in the biosynthesis of alternariol. Furthermore, RNA-Seq assays showed that only expression of the aurovertin gene cluster is much stronger than expression of the housekeeping genes under laboratory conditions, consistent with the observation that aurovertins are the predominant metabolites. Gene expression of the remaining 64 gene clusters for compound backbone biosynthesis was all lower than expression of the housekeeping genes, which partially explained poor production of other secondary metabolites in this fungus.

**Conclusions:**

Our omics data, along with bioinformatics analysis, indicated that *C. arbuscula* NRRL 3705 contains a large number of biosynthetic gene clusters and has a huge potential to produce a profound number of secondary metabolites. This work also provides the basis for development of endophytic fungi as a new resource of natural products with promising biological activities.

## Background

Fungi are important sources of natural product-derived drugs, such as penicillin, cephalosporins, lovastatin and cyclosporin A [1, 2]. Endophytic fungi are those that live in various tissues and organs of healthy hosts at a certain stage or all stages of their life history, and generally do not confer external symptoms to the infected hosts [3]. They can be developed as biopesticides by artificial introduction into other plants and are thus inherited by the host seeds. Endophytic fungi are also gradually attracting scientists’ attention due to its ability to produce natural products, especially some bioactive compounds such as taxol, sequoiatone A and B (antitumor) [4, 5], cryptocandin and cryptocin (antibiotics) [6, 7], peramine, loline, lolitrem B and ergovaline (insecticides) [8, 9], IAA, acetonitrile (plant growth regulators) and subglutinol A and B [10, 11]. However, there is currently little research on the biosynthesis capacity of endophytic fungi and its secondary metabolites, especially at the omics levels, which has limited our understanding and development of these resources.

*Calcarisporium*, a genus of fungi founded by Preuss, is characterized by a transparent, conical, spore-like sporophyte with spores [12]. Most research on this genus has focused on species classification and bio-morphological studies. Some reports also have shown bioactive natural products from the fermentation of this fungal genus, such as 15G256α, 15G256α-2, 15G256β, 15G256β-2 and calcarides A-E [13], suggesting that fungi in this genus might be new promising sources of natural products. However, no details about the genomic information of relevant species in this genus have been reported.

*Calcarisporium arbuscula* is an endophytic fungus from the fruit-bodies of Russulaceae, which displays resistance to other fungi by producing certain antibiotics [14]. It can produce a large number of aurovertin-type mycotoxins as inhibitors of the F0F1-ATP synthase [15, 16], such as aurovertin B as a potential therapeutic against cancer [15], and aurovertin D with strong toxicity towards the root-knot nematode *Meloidogyne incognita* [17]. *C. arbuscula* is also considered a myco-parasite due to its ability to kill the pathogen of coffee plantations - *Hemileia vastatrix* [18]. In addition, the draft genome sequence of this fungus has shown the ability to produce a rich repertoire of natural products, and intriguing compounds with attractive structures and bioactivities were discovered after epigenetic activation [16, 19]. These findings suggested that *C. arbuscula* is of great potential for biological control and new drug development. However, the lack of detailed information about its genome and transcriptome has limited our further understanding and development of this fungus as a representative species of the *Calcarisporium* genus.

Recently, a large number of fungal genome programs have been launched (1000 fungal genomes project, http://1000.fungalgenomes.org) to facilitate the access to more secondary metabolites at the genomic level. Genomic studies have shown that fungi contain a larger number of biosynthetic gene clusters than ever expected for secondary metabolite production [20]. However, most gene clusters are silent under laboratory conditions and the fungi are therefore unable to produce corresponding secondary metabolites.

To further understand this endophytic fungus, particularly its potential in production of secondary metabolites, we report here a the genome sequence of *C. arbuscula* NRRL 3705, which was generated bythe high quality Single Molecule Real-Time (SMRT) sequencing technology. The genome annotation and transcriptome assays revealed that *C. arbuscula* NRRL 3705 harbors many secreted proteins, virulence factors and CAZymes. This genome furthermore contains a large number of gene clusters involved in biosynthesis of secondary metabolites, including aurovertins and other mycotoxins. We demonstrated that low activity of most gene clusters in *C. arbuscula* NRRL 3705 is most likely the result of low levels of transcription, as revealed by RNA-Seq assays. Moreover, the genome information can be further used for comparative genomic studies and discovery of more novel secondary metabolites.

## Results

### Genome sequencing and annotation

**Fig. 1:**
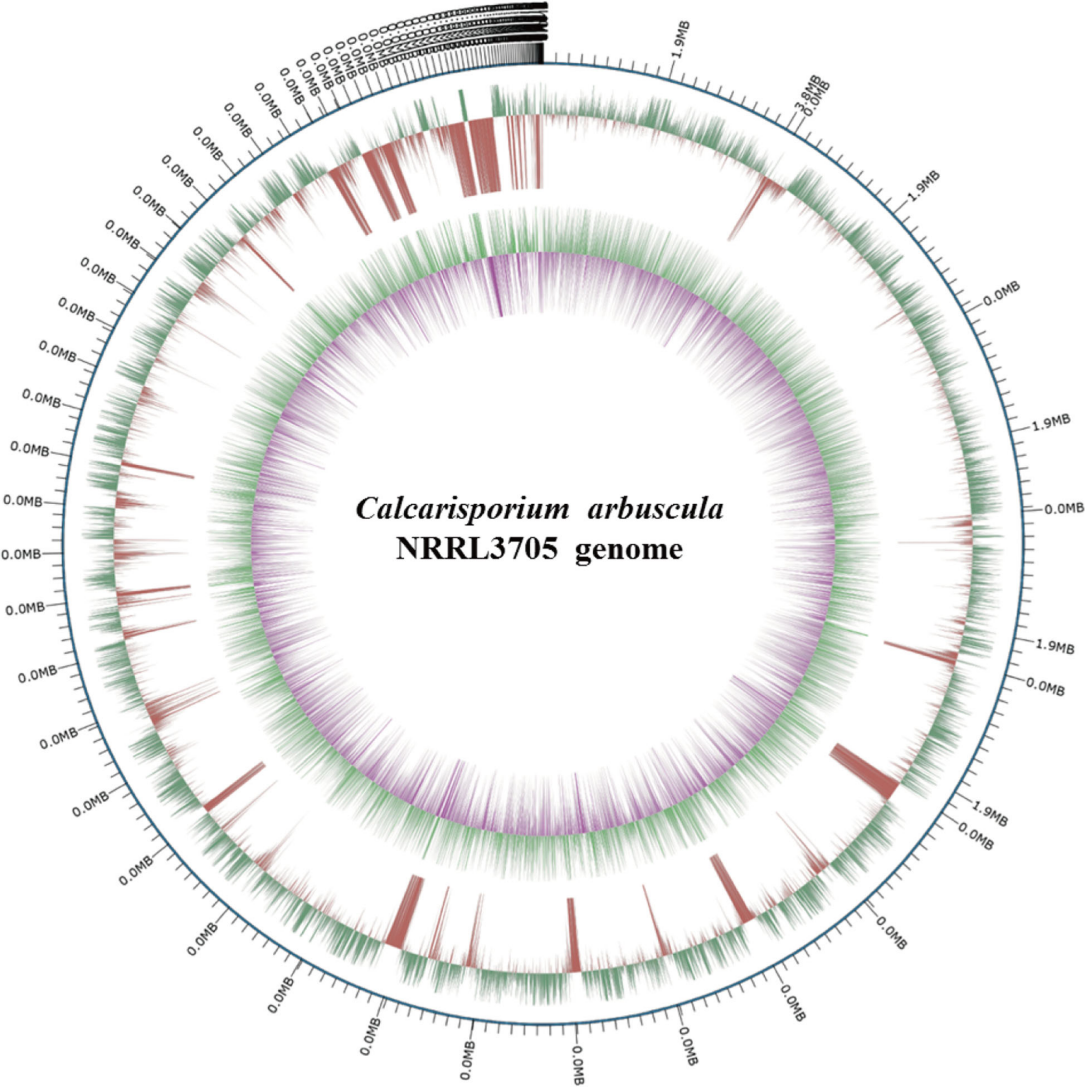
Circos-plot of *C. arbuscula* NRRL 3705. The 91 contigs of *C. arbuscula* NRRL 3705 are displayed by circos-plot (Mb scale). The circos from outside to inside are: (a) 99 contigs; (b) DNA methylations (+); (c) DNA methylations (−); (d) GC content; (e) GC preference

The genome of *C. arbuscula* NRRL 3705 was sequenced by Illumina Miseq technology and third generation sequencing technology (Single-Molecule Real-Time sequencing technology) with over 100X coverage [21]. This method can efficiently decode difficult but important genomic areas, such as methylated regions, repetitive elements and non-coding regions for possible gap-free eukaryotic genome assembly. Sub-read distribution analysis confirmed high quality of the 20-kb library (Additional file 1: Fig. S1). Moreover, we had RNA-Seq results serving as a reference for genome annotation. Combining genomic data and transcriptome analysis make genome assembly and annotation more accurate. The details of data generation are listed in Additional file 2. The completeness of genome assembly and annotation with single copy orthologs test results suggested a well completed annotation set, with 94.1% of the Fungi BUSCOs being present within the RefSeq annotation set, and 4.8% of those fragmented. Details of BUSCO analysis are presented in Additional file 3. The genome was finally assembled with a size of approximately 45.01 Mb, comprising 91 contigs as displayed by circos-plots (Fig. 1) with an N50 length of 1,530,317 bp,which is larger than the genome size of *Calcarisporium sp.* (Table 1) [22]. A total of 10,001 genes were predicted and the average gene length is 1365 bp. The total coding region of *C. arbuscula* is 13.6 Mb, accounting for 30.2% of the entire genome. Statistics analysis for gene length distribution of *C. arbuscula* showed that 752 genes have a length over 2500 bp (Additional file 1: Fig. S2). Notably, compared to other commonly studied filamentous fungi, *C. arbuscula* has a relatively large genome, since most other fungal genomes are less than 40 Mb in size (Table 1) [23–26]. In addition, there are also sections about non-coding proteins (Additional file 2). We also performed RNA-Seq with wild-type strains of *C. arbuscula* and we found 9005 genes being expressed under laboratory conditions. This accounts for 96.72% of total genes in the corresponding genomes (Additional file 4). Based on the fragments per kilobase of transcript per million mapped reads (FPKM) values, we divided the expressed genes into nine tiers (Additional file 4).

**Table 1:** Summary of main genome features of *C. arbuscula* NRRL 3705 and four sequenced fungi

Species	Genome size (Mb)	%GC	Proteins	Ref
*C. arbuscula*	45.01	49.75	10,001	This study
*Calcarisporium sp*	36.8	50.6	15,459	[22]
*Aspergillus nidulans*	30.1	50.32	10,560	[23]
*Aspergillus niger*	33.9	50.36	8592	[24]
*Aspergillus oryzae*	36.7	48.24	12,063	[25]
*Metarhizium robertsii*	39.04	51.49	10,582	[26]

The annotations of 10,001 protein-encoding genes are reported in Additional file 5. Among all coding genes, 9397 genes could be annotated by various databases (Additional file 1: Fig. S3). Using the Non-Redundant Protein Database for protein annotation, we found that 8816 genes were protein-encoding genes (Additional file 1: Fig. S3), which account for 88.16% of the total coding genes. KEGG analysis revealed that the products of most genes are involved in metabolism: carbohydrates (≈ 321 proteins), amino acids (≈ 252 proteins), or lipids (≈ 161 proteins) (Additional file 1: Fig. S4). These data suggested that *C. arbuscula* may produce a large number of enzymes involved in its rich metabolic processes. Gene Ontology (GO) functional classification of *C. arbuscula* (Additional file 1: Fig. S5) also showed that most genes are involved in catalytic activity (≈ 3649 proteins / strain) and metabolic processes (≈ 3654 proteins / strain). In addition, Eukaryotic Orthologous Groups (KOG) functional classification showed that many genes are involved in posttranslational modifications (Additional file 1: Fig. S6).

### Taxonomy

*C. arbuscula* NRRL 3705 is an endophytic fungus in fruit-bodies of Russulaceae, producing aurovertin-type mycotoxins that are potent against F0F1-ATPase and breast cancers [14–16]. According to fungus taxonomy, it belongs to *Calcarisporium*, Hypocreales, Pezizomycotina, Ascomycota. Spores of *C. arbuscula* NRRL 3705 develop after culturing on potato dextrose agar (PDA) medium for 5 days at 25 °C. The filamentous fungus displays high sporulation and the conidial heads are yellow (Additional file 1: Fig. S7).

**Fig. 2:**
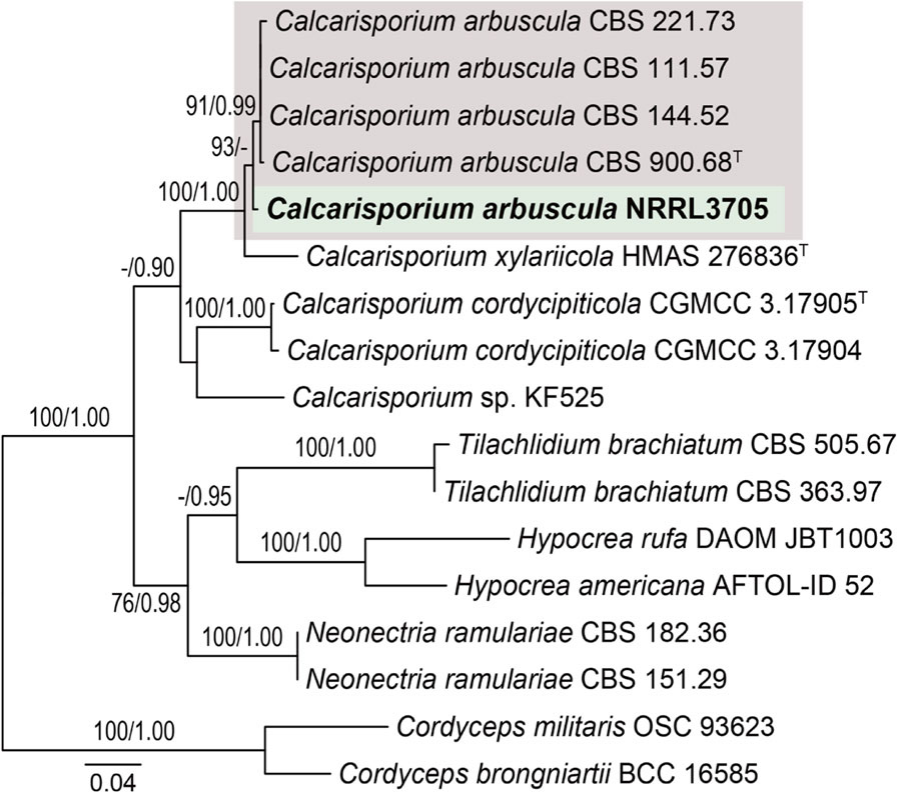
Phylogenetic and synteny analysis of *C. arbuscula* NRRL 3705 with other fungal species. Multilocus phylogenetic analysis of *Calcarisporium* based on a combined SSU, ITS, LSU, TEF and RPB2 data set. The tree is rooted with *Cordyceps militaris* and *Cordyceps brongniartii*. Bootstrap values higher than 50% from RAxML (BSML) (left) are given above the nodes. Bayesian posterior probabilities greater than 0.90 are indicated (BYPP) (right). ^T^ indicates type

The phylogenetic analysis performed in this study used several reference genes (ITS, SSU, LSU, TEF and RPB2) and revealed the close relationship between the sequenced strain and other strains. The multilocus analysis was performed on our isolate with 17 reference strains (NCBI accession number available in Additional file 6). For species delimitation, the aligned sequences matrix of (ITS, SSU, LSU, TEF and RPB2) sequences data for *Calcarisporium* and for *Cordyceps militari*s and *C. brongniartii* as outgroup taxa. The phylogenetic tree was constructed with maximum likelihood and Bayesian analysis and resulted with high bootstrap values (Fig. 2). This tree illustrated that *C. arbuscula* NRRL3750 was most closely related to *C. arbuscula* 111.57 and *C. arbuscula* 144.52.

### Repetitive elements

**Fig. 3:**
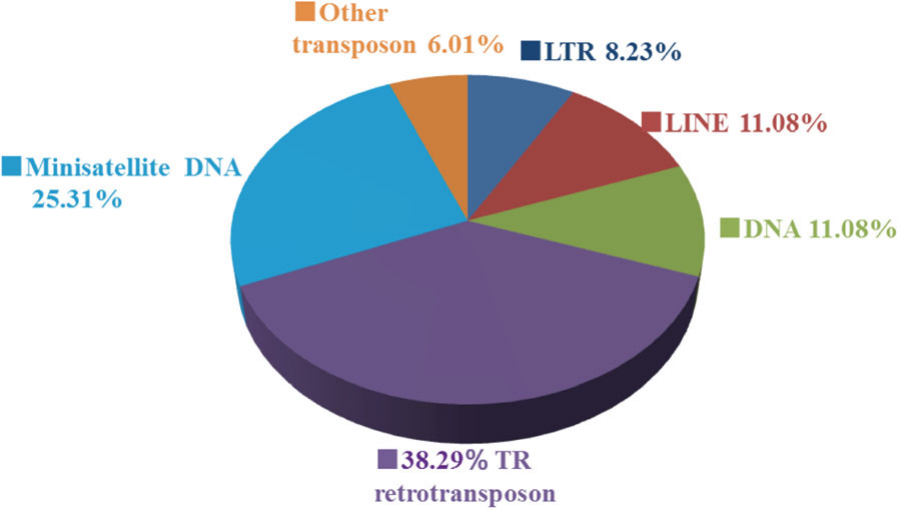
Repeat elements of *C. arbuscula* NRRL 3705. The percentage of different types of repetitive sequences in the *C. arbuscula* genome

Repeated sequences play an important role in maintaining the spatial structure of chromosomes, gene expression regulation and genetic recombination of fungi [27]. A total of 1,387,508 bp of repeat sequences were identified in the *C. arbuscula* genome, including LTR retrotransposons, DNA transposons, long interspersed repeated elements (LINEs), tandem repeat sequences (TR) and mini-satellite DNA. Interestingly, the majority of repetitive sequences (63.6%) are tandem repeat sequences, whereas the dispersed repetitive-sequence just accounts for 30.39%. Notably, the highest percentage of all repeat sequences is TR, at 38.29% (Fig. 3).

### Predicted candidate secreted effectors involved in virulence and pathogenicity

Secreted effectors play critical roles in virulence and pathogenicity [28, 29]. The signal peptide prediction tool SignalP was used to identify proteins containing the cross-membrane structures [30]. In total, there were 762 possible secretion proteins identified, accounting for 7.62% of all predicted proteins (10,001). Based on alignment analysis of all predicted proteins against the pathogen-host interaction (PHI) database [31], 228 out of 10,001 (2.29%) predicted proteins were related to pathogenicity, of which 21 (0.21%) putative PHI-related proteins were potential secreted effectors.

**Fig. 4:**
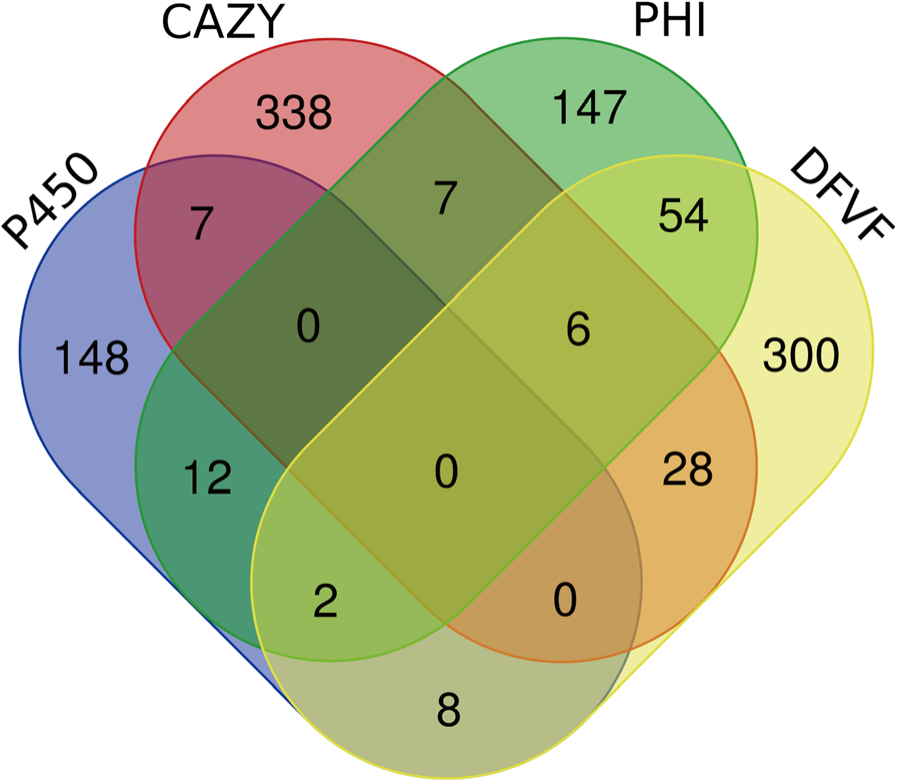
Venn-plot showing the intersections among the secreted PHI proteins (green), secreted DFVF (yellow), secreted CYP450 enzymes (purple), and secreted CAZymes (red)

After whole proteome BLAST against the database of fungal virulence factors (DFVF) [32], 398 out of 10,001 (39.8%) predicted proteins encoded within the *C. arbuscula* genome were identified to share identity with proteins implicated in virulence, of which 63 (0.63%) putative DFVF-related proteins were predicted to be secreted. Furthermore, 62 of these secreted proteins were predicted to be involved in pathogen-host interactions (Fig. 4).

P450 enzymes not only participate in the production of important internal metabolites, but also play an important role in adaptation to different environments by modifying harmful chemicals [33]. By BLASTP, the amino acid sequences of all *C. arbuscula* proteins were compared to the Fungal Cytochrome P450 Database (FCPD), 177 out of 10,001(1.77%) was identified as putative CYP450 enzymes, part of which are involved in fungal virulence factor and pathogen-host interaction (Fig. 4).

### Carbohydrate-active enzymes

Carbohydrate-active enzymes (CAZy) play an important role in carbohydrate degradation, modification and biosynthesis in fungi [34]. CAZy is also a Carbohydrate-Active enZYmes Database [35], a specialized database of carbohydrate enzymes, which includes a family of related enzymes that catalyze the degradation, modification, and biosynthesis of carbohydrates. CAZy’s are divided into five main categories: Glycoside Hydrolase (GH) [36], Glycosyl Transferase (GT) [37], Polysaccharide Lyases (PL) [38], Carbohydrate Esterases (CE) [39], and Oxidoreductase (Auxiliary Activitie, AA). In addition, it also contains Carbohydrate-Binding Module (CBM) proteins [40]. In the *C. arbuscula* genome, 386 proteins were identified as CAZymes,part of which are involved in pathogen-host interactions (Fig. 4). The highest proportion (62.29%) of all CAZymes belonged to the GH category (Additional files 1: Fig. S8). Based on genomic information, we compared the potential for hydrolysis with eight *Aspergillus* species. Notably, *C. arbuscula* NRRL 3705 contains a large amount of glycoside hydrolases GH18 and GH2, more than found in other fungi (Additional file 7).

### Secondary metabolite biosynthetic gene clusters in *C. arbuscula* NRRL 3705

The secondary metabolites of fungi constitute a rich resource of bioactive compounds with potential pharmaceutical values as antibiotics, cholesterol-lowering drugs and antitumor drugs [1]. Interestingly, genes encoding the biosynthetic pathway responsible for the production of such secondary metabolites are often spatially clustered together; such a compendium of genes is referred to as a ‘secondary metabolite biosynthesis gene cluster’ [41]. Based on profile hidden Markov models of genes that are specific for certain types of gene clusters and antiSMASH 4.0, we identified 65 gene clusters for secondary metabolites in *C. arbuscula* NRRL 3705. Among them, 23 and 12 gene clusters containing genes encoding polyketide synthases (PKS) and non-ribosomal peptides synthases (NRPS), respectively, were identified. In addition, there are gene clusters for terpenes, PKS/NRPS hybrids, indoles and other types of natural products (Additional file 8). Some of these gene clusters are highly similar to known gene clusters (Table 2).

**Table 2:** Prediction of possible secondary metabolites of gene clusters in *C. arbuscula* NRRL 3705

Cluster	Type	Smiliarty to known clusters
Cluster1	other	
Cluster2	terpene	
Cluster3	t1pks-nrps	
Cluster4	terpene	
Cluster5	t1pks-terpene	Sordarin (32% of genes show similarity)
Cluster6	nrps	
Cluster7	nrps	
Cluster8	terpene	
Cluster9	t1pks	
Cluster10	t1pks	
Cluster11	t1pks	
Cluster13	t1pks	
Cluster14	nrps	
Cluster16	terpene	
Cluster17	t1pks-nrps2385	
Cluster18	nrps	
Cluster19	terpene	
Cluster20	nrps	
Cluster21	nrps	
Cluster22	indole-t1pks	
Cluster23	t1pks	Citreoviridin (40% of genes show similarity)
Cluster24	t1pks	
Cluster25	terpene	
Cluster26	t1pks-nrps	Aculeacin A (NRPS 100% only)
Cluster27	t1pks	
Cluster28	lantipeptide	
Cluster29	terpene	
Cluster30	nrps	
Cluster31	t1pks	
Cluster32	t1pks	
Cluster33	nrps	Dimethylcoprogen (100% of genes show similarity)
Cluster34	t1pks	
Cluster35	t1pks	Alternariol (100% of genes show similarity)
Cluster36	nrps	
Cluster37	nrps	Destruxin (66% of genes show similarity)
Cluster38	t1pks	
Cluster40	t1pks-nrps	
Cluster41	t1pks-nrps	Citrinin(18% of genes show similarity)
Cluster42	terpene	Copalyl_diphosphate(28% of genes show similarity)
Cluster43	t1pks-nrps	Leucinostatins (10% of genes show similarity)
Cluster44	t1pks	Alternariol (100% of genes show similarity)
Cluster45	t1pks	
Cluster46	t1pks	
Cluster47	t1pks	
Cluster48	t1pks	
Cluster51	t1pks	
Cluster52	t1pks-nrps	
Cluster53	t1pks	
Cluster54	indole	
Cluster55	t1pks	
Cluster56	nrps	
Cluster57	terpene	
Cluster58	nrps	Isoflavipucine(12% of genes show similarity)
Cluster59	other	Destuxin (66% of genes show similarity)
Cluster60	t1pks	Aflatoxin (46% of genes show similarity)
Cluster61	t1pks	
Cluster62	t1pks	
Cluster63	terpene	
Cluster64	terpene	
Cluster65	other	

### Aurovertin biosynthetic gene cluster

**Fig. 5:**
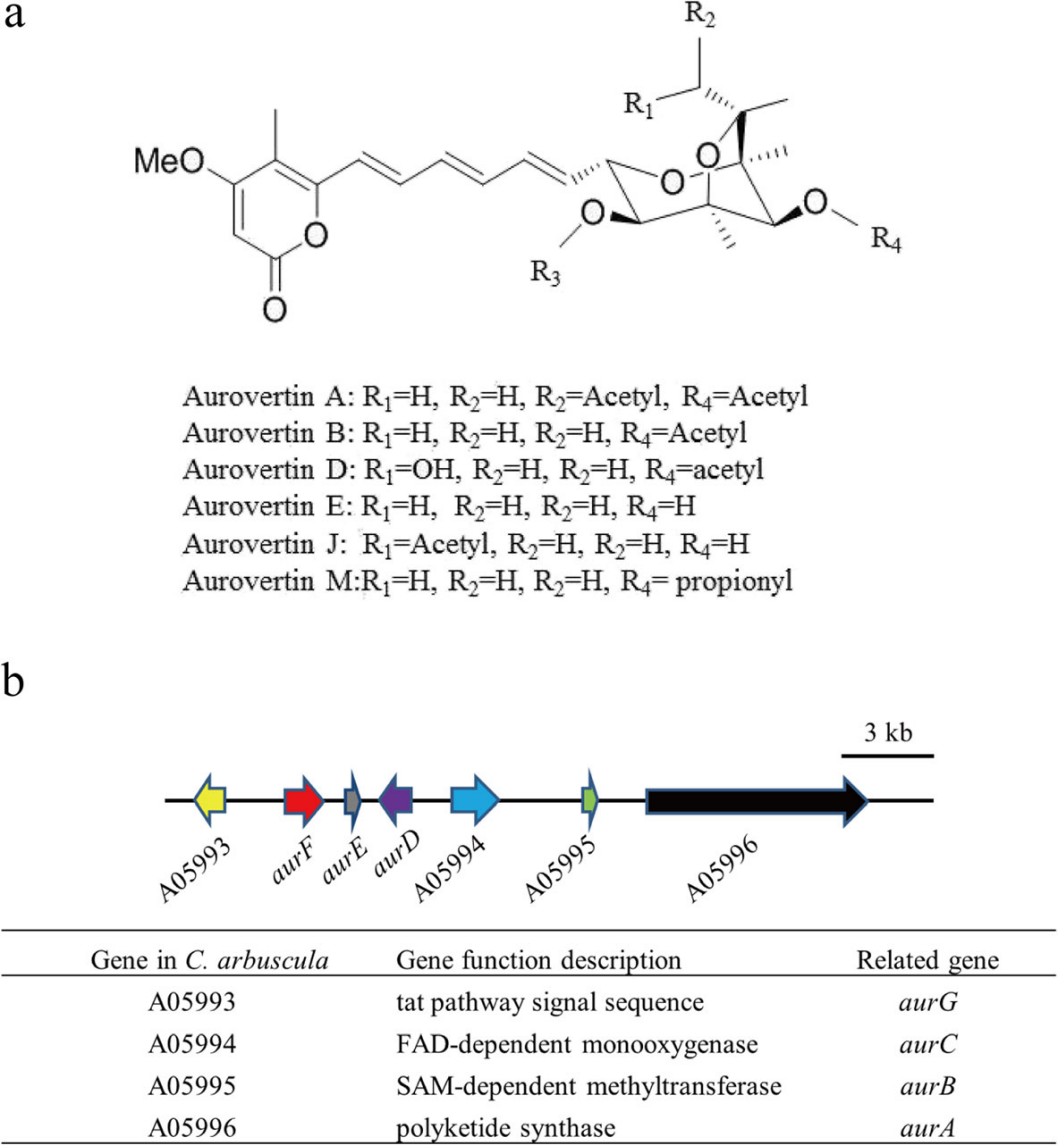
Aurovertin biosynthesis in *C. arbuscula* NRRL 3705. **a.** Chemical structures of aurovertins. **b.** Schematic representation of the putative aurovertin gene cluster (cluster 23) and the description of each gene in a gene cluster

Aurovertins are a class of toxic polyketides harboring a unique structure of a 2, 6-dioxabicyclo-[3.2.1]-octane (DBO) ring system and a conjugated α-pyrone moiety [16, 42]. Due to the unusual polyketide-derived structure, aurovertins have been shown to have potent antiviral, antitumor and antibacterial activities. *C. arbuscula* is capable of predominantly producing aurovertins (Fig. 5a), and the biosynthetic gene cluster for these mycotoxins has been identified [16]. In addition, an LC-MS analysis was performed on a methanol extract obtained from a 5-days-old culture of *C. arbuscula* NRRL3705 grown on a PDA plate at 25 °C (Additional files 1: Fig. S9 and Fig. S10). We have also performed a phylogenetic analysis of the aurovertin-related gene cluster of different strains (Additional files 1: Fig. S11). The aurovertin biosynthetic gene cluster was mainly composed of seven genes, including *aurA, aurB, aurC, aurD, aurE, aurF* and *aurG*. However, some genes were missing in the cluster after genome annotation by automatic bioinformatics.

According to antiSMASH 4.0, there are totally 4 genes in gene cluster 23 (aurovertin biosynthetic gene cluster). These genes encode a PKS (A05996), a SAM-dependent methyltransferase (A05995), a FAD-dependent monooxygenase (A05994), and an acetyltransferase (A05993) (Fig. 5b). We found that there is a 7 kb spacer between gene A05993 and A05994, which was re-predicted by the web-based software Softberry (http://www.softberry.com/)and we found that this spacer contains three known genes: *aurD*, *aurE* and *aurF*. This is consistent with the gene cluster reported previously [16]. This also indicates that there are certain defects in genome sequencing and automatic NR annotation.

### Other SM clusters for mycotoxin biosynthesis

**Fig. 6:**
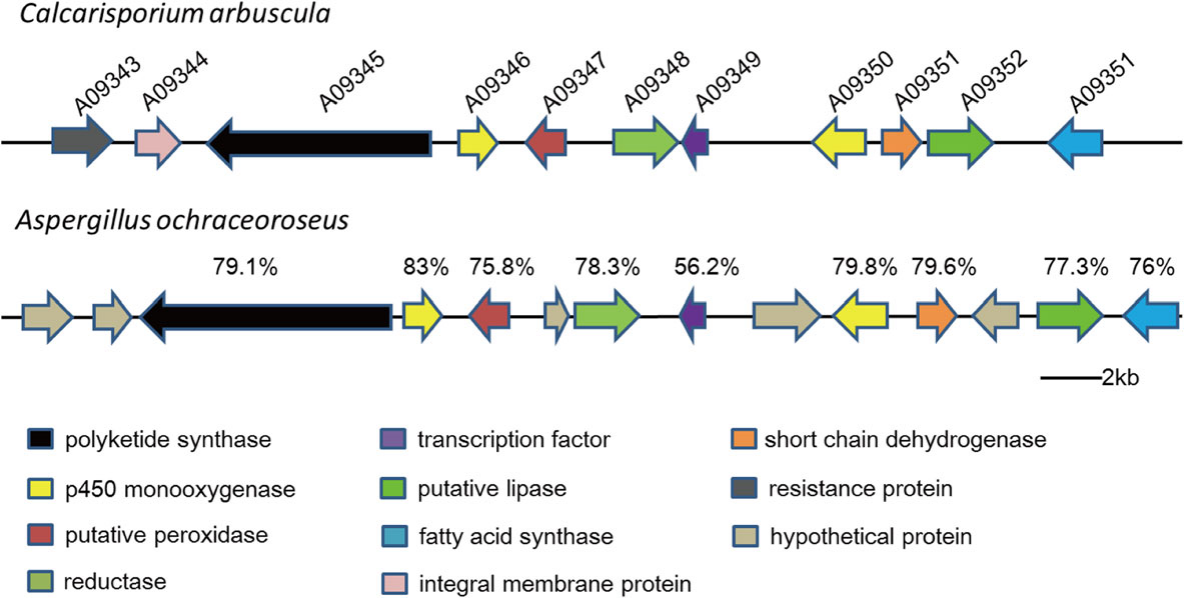
The putative aflatoxin biosynthetic gene cluster of *C. arbuscula* NRRL 3705 found in this study and the comparison of this cluster with the aflatoxin cluster reported for *Aspergillus ochraceoroseus*. The identity of each homolog to *C. arbuscula* NRRL 3705 counterparts is shown

Based on antiSMASH predictions, *C. arbuscula* NRRL 3705 has the potential to produce a variety of mycotoxins. Aflatoxin (AFT), a class of toxic secondary metabolites originally produced by *Aspergillus parasiticus*, is highly toxic, carcinogenic, mutagenic and teratogenic [43]. Cluster 60 is composed of 15 genes and contains a PKS (A09345), a putative ketoreductase (A09348), a transcription factor (A0934) and two cytochrome P450 monooxygenases (A09346 and A09350). PKS (A09345) of cluster 60 shows high sequence identity with AflC (a polyketide synthase involved in aflatoxin biosynthesis) of *A. ochraceoroseus* (protein coverage: 98%; identity: 79%). Moreover, the two cytochrome P450 monooxygenases of cluster 60 show highest sequence identity with AflV (protein coverage: 99%; identity: 83%) and AflG (protein coverage: 96%; identity: 79.8%) of *A. ochraceoroseus* (Fig. 6). These in silico data suggested that *C. arbuscula* potentially produces compounds and derivatives structurally related to aflatoxin.

**Fig. 7:**
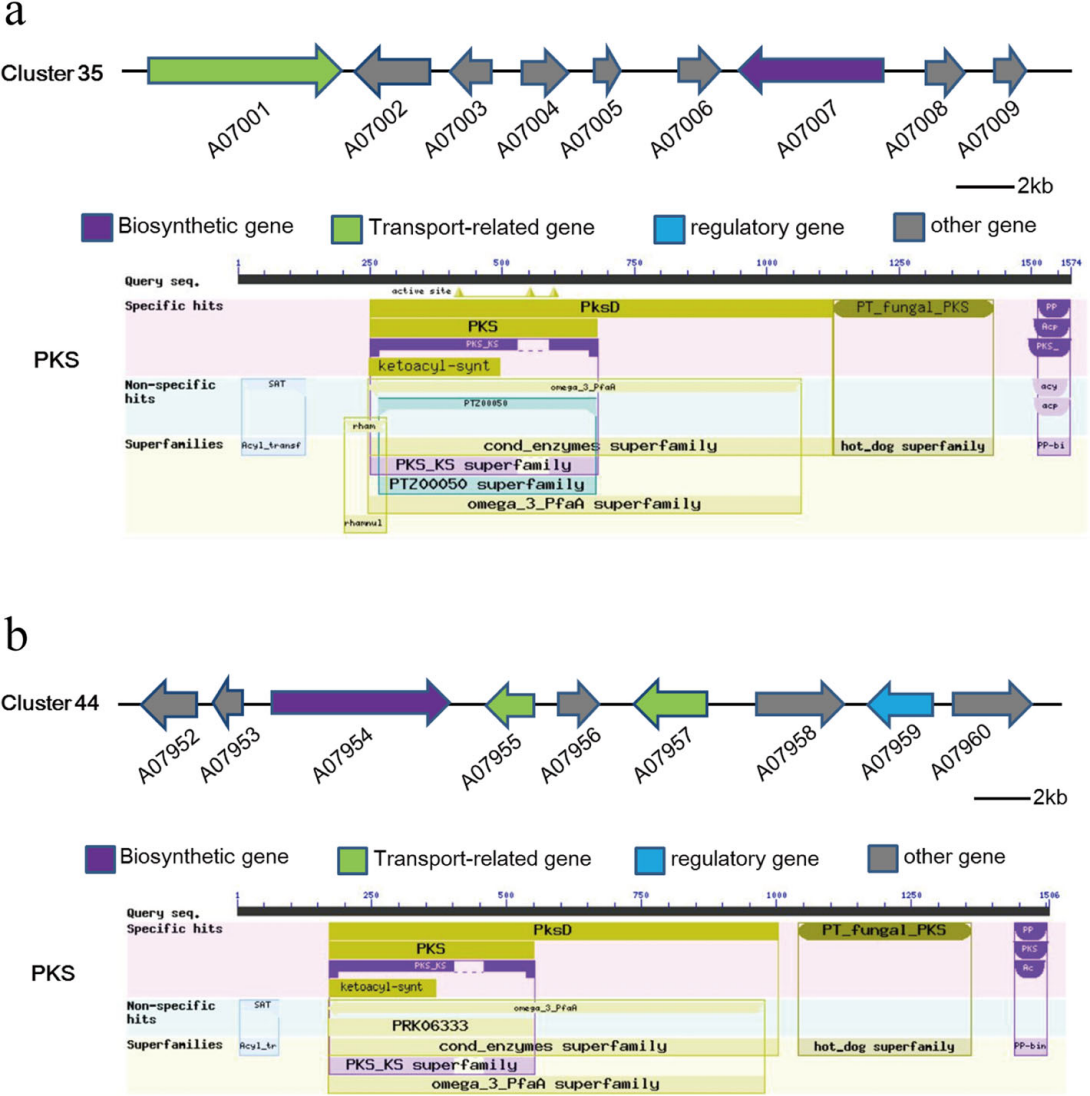
Two AOH biosynthesis-related gene clusters for *C. arbuscula* NRRL 3705. **a** Cluster 35, with 7 genes. **b** Cluster 44, with 7 genes. The PKS and domains were determined by the BLAST searches against the CDD database

In addition, we found that *C. arbuscula* has the potential to produce alternariol (AOH) [44]. Alternariol, a secondary metabolite produced by *Alternaria* and other fungi, is harmful to animals and plants. One polyketide synthase (PKS 19) from *Parastagonospora nodorum* has shown to be responsible for AOH production in this fungus. Surprisingly, we found two candidate gene clusters (cluster 35 and cluster 44) with high similarity to the alternariol biosynthetic gene cluster from *P. nodorum* SN15. Cluster 35 is composed of 9 genes. The backbone gene encodes a PKS (A07007), while other genes encode an NAD^+^-binding protein (A07006), an acyl-CoA-acyltransferase (A07005), an aldehyde dehydrogenase (A07004), an integral membrane (A07003), an arginosuccinate synthetase (A07002), an ABC transporter (A07001), a putative capsule polysaccharide biosynthesis protein (A07008) and a transcriptase (A07009) (Fig. 7a). Cluster 44 is also composed of 9 genes that encode a PKS, four putative signal sequence proteins and a transcription factor (Fig. 7b). The PKS from cluster 35 shares higher identity with PKS 19 (72%) than with the PKS from cluster 44 (44%). Considering that the cluster for AOH biosynthesis in *P. nodorum* contains one PKS and four tailoring enzymes (*O*-methyl transferase OmtI, monooxygenase MoxI, short chain dehydrogenase like protein SdrI and an estradiol dioxygenase DoxI), cluster 35 is more likely responsible for the biosynthesis of AOH. In contrast, cluster 44 lacks multiple enzymes, as shown above. Therefore we hypothesize that cluster 35 is more likely the putative SM cluster for biosynthesis of AOH. However, this needs to be validated by further genetic and biochemical analysis.

The non-ribosome polypeptide synthase is responsible for the synthesis of peptide secondary metabolites, such as surugamides and ferricrocin [45, 46]. In *C. arbuscula*, 12 putative NRPS genes were found. According to antiSMASH and MIBiG, cluster 37 and cluster 59 show sequence similarity with the biosynthetic gene cluster of destruxin, a secondary metabolite of non-ribosomal cyclic hexapeptides with insecticidal and pharmaceutically active activities (Additional file 8).

### Gene clusters for biosynthesis of other secondary metabolites

In the *C. arbusclua* genome, seven hybrid NRPS / PKS gene clusters were also predicted. Among them, cluster 26, cluster 41 and cluster 58 show similarity to the gene clusters of aculeacin A, citrinin and isoflavipucine, respectively (Additional file 8) [47–49]. Moreover, we also predicted eleven terpene genes, whose products remain to be determined. In addition to the PKS, NRPS, and hybrid NRPS/PKS gene clusters, we also identified 12 gene clusters likely to produce indoles, one hybrid indole-t1PKS and one hybrid t1PKS-terpene. Overall, we have performed a thorough comparative analysis and shown as much information on gene clusters as possible after antiSMASH and gene cluster homology comparisons. We have shown some predicted chemical structures of natural products synthesized by the corresponding gene clusters, while most gene clusters share low sequence identity with others, or produce unknown natural products, which also raises high interests for further investigation of these gene clusters experimentally.

### Gene cluster expression by RNA-Seq analysis

**Fig. 8:**
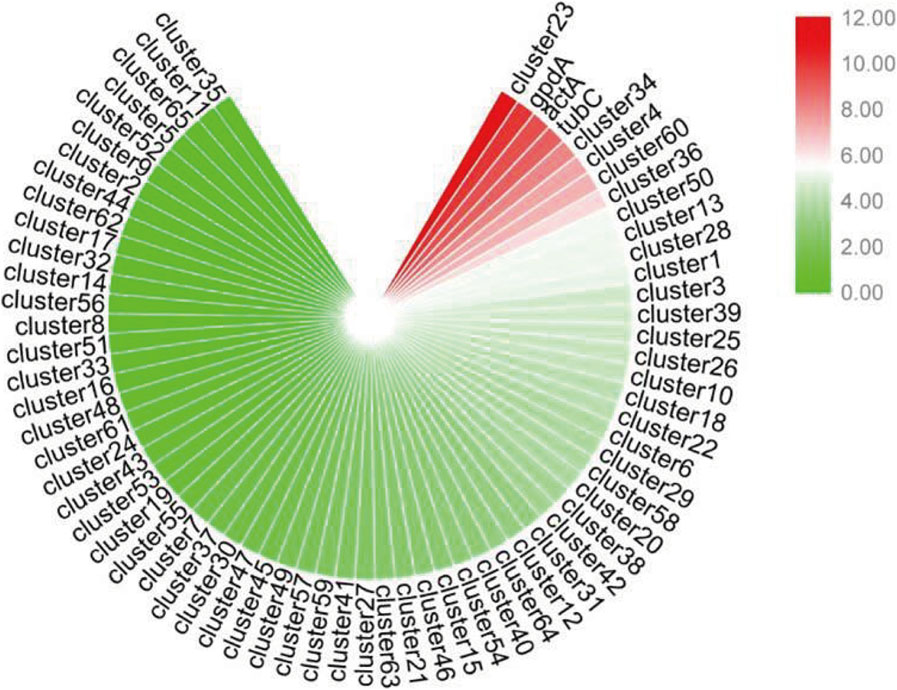
Comparison the backbone gene experssion of every gene cluster in *C. arbuscula* NRRL 3705.Heat map of gene expression for the every gene cluster in *C. arbuscula* NRRL 3705*.* The house-keeping genes *actA, tubC* and *gpdA* are the reference genes

Although *C. arbuscula* contains a large number of biosynthetic gene clusters, this fungus rarely produces secondary metabolites, except aurovertins [16]. It is very likely that under laboratory culture conditions, expression of the core genes of most gene clusters is low. Therefore, RNA-Seq assays were performed and gene expression was evaluated based on FPKM from RNA-Seq. Using three housekeeping genes (*gpdA*, *tubC* and *actA*) as reference genes, we found that only the PKS gene in cluster 23 (aurovetin biosynthetic gene cluster) was expressed at the highest level in core genes of all gene clusters (Fig. 8). These results confirmed that most gene clusters are expressed at low levels or silenced.

In addition, nine gene clusters contain pathway-specific transcription factors (Additional file 8), which provides the possibility to activate these gene clusters by overexpression of these transcription factors to obtain secondary metabolites.

## Discussion

*Calcarisporium arbuscula*, an endophytic fungus from the fruit bodies of Russulaceae, produces a variety of secondary metabolites [14–17], but no a comprehensive survey of the genome and transcriptome of this endophytic fungus has been previously carried out in *C. arbuscula* up to now.

To further understand this endophytic fungus *C. arbuscula*, particularly its potential in production of secondary metabolites, in this study, we sequenced the genome of the important endophytic fungus, *C. arbuscula*. The genome of this fungus is over 45 Mb in size and comprises 10,001 predicted genes in this fungus.

It is worth noting that two of the enriched family is glycoside GH18 family and GH2 family in *C .arbuscula* (Additional file 7). The fungal glycoside hydrolase GH18 is mainly responsible for remodeling and recovery of the fungal cell wall and other cell wall degrading enzymes [50]. The fungal glycoside hydrolases GH2 can specifically hydrolyze the β-glycosidic bond between d-glucuronidose and aglycon, and has important applications in the diagnosis and drug development of metabolic diseases [51]. We hypothesize that the expansion of GH18 family and GH2 family in *C. arbuscula* genome may contribute to help the host to resist the attack of other fungi. Endophytic fungus *C. arbuscula* may also be a mycorrhizal helper fungus, which can promote the growth of mycorrhizal fungi and colonize the roots of the host plant to form a mycorrhizal symbiotic structure, thereby promoting plant growth.

*C. arbuscula* is an endophytic filamentous fungus, estimated to have 65 BGCs, but most of them are silenced [16, 19]. Although some gene clusters are expressed at the transcription levels by RNA-Seq, we have not detected their corresponding products, and it is likely that the translation level of the main proteins in the gene clusters is still low. These silent gene clusters are a valuable resource, and later we can try to activate them in different ways to obtain more novel natural products.

In summary, we report a high-quality genome sequence of *C. arbuscula* by SMRT sequencing method. By the genome assembly and annotation, we hypothesize that *C. arbuscula* NRRL 3705 contains a large number of biosynthetic gene clusters and has a huge potential to produce a profound number of secondary metabolites. This work helps us to understand its capacity to biosynthesize secondary metabolites and will lay the foundation for the development of this precious resource.

## Conclusions

In this study, we reported the high-quality genome sequence of *C. arbuscula* NRRL 3705. Phylogenetic tree analysis showed that this fungal strain is unique from the same species. This fungus contains 65 gene clusters involved in biosynthesis of a variety of secondary metabolites, including aurovertins and other putative mycotoxins. We also demonstrated that most gene clusters are silenced or display low expression levels, most likely due to the low levels of transcription, as shown by RNA-Seq assays. The genomic and transcriptomic survey of *C. arbuscula* NRRL 3705 will help us in further development of this fungus for discovery of new bioactive natural products.

## Methods

### Fungal culture and genomic DNA extraction

The isolated spores of *C. arbuscula* NRRL 3705 were inoculated on potato dextrose agar (PDA) (Sigma) medium. Fungal mycelia were grown at 25 °C for 5 days, collected and grounded into powder with liquid nitrogen. Genomic DNA was extracted with the cetyltrimethylammonium bromide (CTAB) method [52]. The harvested DNA was detected by agarose gel electrophoresis and quantified by Nanodrop.

### Sequencing and assembly

Libraries for single-molecule real-time (SMRT) sequencing were constructed with an insert size of 20 kb using the SMRT bell TM Template kit, version 1.0. Briefly, the process includes blunt-end ligation, SMRTbell Templates purification with 0.45X AMPure PB Beads, size-selection using the BluePippin System, and DNA damage repair after size-selection. Finally, the library quality was assessed on the Qubit® 2.0 Fluorometer (Thermo Scientific) and the insert fragment size was analyzed by Agilent 2100 (Agilent Technologies).

A total amount of 1 μg of DNA per sample was used as input for DNA sample preparation. Sequencing libraries were generated using NEBNext® Ultra™ DNA Library Prep Kit for Illumina (NEB, USA) following manufacturer’s recommendations and index codes were added to attribute sequences to each sample. Briefly, the DNA sample was fragmented by sonication to a size of 350 bp, then DNA fragments were end-polished, A-tailed, and ligated with the full-length adaptor for Illumina sequencing with further PCR amplification. Last, PCR products were purified (AMPure XP system) and libraries were analyzed for size distribution by Agilent2100 Bioanalyzer and quantified using real-time PCR [53].

The whole genome of *C. arbuscula* was sequenced using the PacBio Sequel platform and Illumina NovaSeq PE150 at the Beijing Novogene Bioinformatics Technology Co., Ltd. Data was processed according to the method described by Yuan et al. [21, 54, 55].

### Genome annotation

Genome component prediction includes the prediction of the coding gene, repetitive sequences and non-coding RNA. The available steps were as follows:

For fungi, by default, using the Augustus 2.7 program to retrieve the related coding genes. If homology reference gene sequences and transcript sequencing data were provided,a complete annotation pipeline, PASA, as implemented at the Broad Institute, involves the following steps: (A) ab initio gene finding using a selection of the following software tools: GeneMarkHMM, FGENESH, Augustus, and SNAP, GlimmerHMM. (B) Protein homology detection and intron resolution using the GeneWise software and the uniref90 non-redundant protein database. (C) Alignment of known ESTs, full-length cDNAs, and most recently, Trinity RNA-Seq assemblies to the genome. (D) PASA alignment assemblies based on overlapping transcript alignments from step (C). (E) Use of EVidenceModeler (EVM) to compute weighted consensus gene structure annotations based on the above (A, B, C, D). (F) Use of PASA to update the EVM consensus predictions, adding UTR annotations and models for alternatively spliced isoforms (leveraging D and E). 2) The interspersed repetitive sequences were predicted using the RepeatMasker (http://www.repeatmasker.org/). The tandem Repeats were analyzed by the TRF (Tandem repeats finder).3) Transfer RNA (tRNA) genes were predicted by the tRNAscan-S.

BUSCO (Benchmarking Universal Single-Copy Orthologs) software was used to assess the completeness of genome assembly and annotation with single-copy orthologs. BUSCO v3.0 was run on the scaffolded genome assembly (using “-m genome”). The lineage dataset of BUSCO was fungi_odb9 (Creation date: 2016-02-13, number of species: 85, number of BUSCOs: 290) [55].

Genome annotation was performed based on de novo prediction and transcriptome-assisted gene prediction. Seven databases were used to predict gene functions, including GO (Gene Ontology), KEGG (Kyoto Encyclopedia of Genes and Genomes), KOG (Clusters of Orthologous Groups), NR (Non-Redundant Protein Database databases), TCDB (Transporter Classification Database), P450, and Swiss-Prot. A whole genome BLAST search (e-value less than 1e-5, minimal alignment length percentage larger than 40%) was performed against above seven databases. The secretory proteins were predicted by the Signal P database. For pathogenic fungi, we added the pathogenicity and drug resistance analysis. We used PHI (Pathogen Host Interactions), DFVF (database of fungal virulence factors) to perform the above analysis. Carbohydrate-Active enzymes were predicted by the Carbohydrate-Active enZYmes Database. BLAST alignment of predicted genes with various functional databases (BLASTP, e-value ≤1e-5); BLAST result filtering: For each BLAST result of the sequence, select the highest score alignment (default identity > = 40%, coverage > = 40%) for annotation of *C. arbuscula*.

### PCR amplification and DNA sequencing

Sequences of ITS and partial LSU ribosomal RNA, partial small-subunit (SSU) ribosomal RNA, translation elongation factor 1 alpha (TEF1-α), and the second largest subunit of RNA polymeraseII (RBP2) were amplified by polymerase chain reaction (PCR) with the primer pairs ITS1–ITS4, LR5F–LROR, NS1–NS4, EF983F–EF2218R and RPB2-5F–RPB2-7cR, respectively. Each amplification reaction included 2 mM of each dNTP, 0.4 mM of each primer, 1 U of KOD FX (Takara, China), 2 μL of genomic DNA solution, 2 × KOD FX buffer in 50 μL reaction volume. A typical reaction included an initial denaturation at 94 °C for 5 min; followed by 30 cycles of denaturation at 98 °C for 10s, annealing at 52 °C for 30 s, extension at 68 °C for 60 s and a final extension at 68 °C for 10 min. Automated sequencing was performed by TsingKe Biological Technology (Hangzhou, China).

### Phylogenetic analysis

The SSU, ITS, LSU, TEF and RPB2 data sets of *Calcarisporium* species determined from recent studies [12], were downloaded from GenBank (Additional file 3) and used in the phylogenetic analysis. Single and combined genes were analysed using maximum likelihood (ML) performed in RAxML implemented in raxmlGUI v.1.3 with rapid bootstrap analysis with 1000 replicates. For Bayesian analyses, the posterior probabilities were determined by Markov chain Monte Carlo sampling (MCMC) in MrBayes v3.2 based on the models from MrModeltest.

PKS sequences were aligned by BLASTN to obtain higher homology sequences in different stranis. Phylogenetic tree about aurA was generated with MEGA7.0 based on Neighbor-Joining method [56]. Bootstrap values were calculated from 500 replications of the bootstrap procedure using phylogeny.fr and added to the phylogenetic tree.

### Repetitive sequences

Repetitive sequences were predicted with RepeatMasker software (version 4.0.5) and Tandem Repeats Finder software [57].

### Biosynthetic gene cluster prediction

Gene clusters were predicted by the web-based software antiSMASH database (antibiotics and Secondary Metabolite Analysis 4.0) [41]. The core genes were annotated using stand-alone BLAST against Swiss-Prot database. Gene cluster domain prediction can be obtained by PKS/NRPS Analysis web-site (http://nrps.igs.umaryland.edu/).

### LC-MS analysis

LC–MS analysis was performed in an Agilent 1200HPLC system (Agilent, Santa Clara, CA, United States) and a Termo Finnigan LCQDeca XP Max LC/MS system (Termo Finnigan, Waltham, MA, United States). Poroshell 120 SB C18 was used as the column, H2O (containing 0.1% formic acid) and acetonitrile (containing 0.1% formic acid) were used as the mobile phase A and B performing a linear gradient from 30 to 100% (v/v) B over 30 min.

### Transcriptome analysis

Transcriptome analysis was performed according to the method of Chen et al. [54]. All three RNA samples were prepared from *C. arbuscula* mycelia as above and subjected to RNA-Seq on the Illumina HiSeq 2000 platform (Illumina, San Diego, CA, USA). *C. arbuscula* wild type was grown on solid PDA media (sigma) at 25 °C for 5 days. Mycelia were collected. Mycelium was sent to the company for testing after being treated with liquid nitrogen. FPKM (fragments per kilobase of transcript per) value was used to evaluate gene expression, and the upper-quartile algorithm was used to correct the gene expression.

## Supplementary information

**Additional file 1.**

**Additional file 2.**

**Additional file 3.**

**Additional file 4.**

**Additional file 5.**

**Additional file 6.**

**Additional file 7.**

**Additional file 8.**

## Data Availability

This Whole Genome Shotgun project has been deposited at NCBI under the accession WBSA00000000. The version described in this paper is version WBSA01000000. The BioProject accession number is PRJNA574730 and the BioSample accession number is SAMN12868444. The data is available at the following URL: https://www.ncbi.nlm.nih.gov/nuccore/WBSA00000000.1/.

## Abbreviations

SMRT: Single Molecule Real-Time
antiSMASH: Antibiotics & Secondary Metabolite Analysis Shell
BLAST: Basic local alignment search tool
GO: Gene Ontology
KEGG: Kyoto Encyclopedia of Genes and Genomes
KOG: Clusters of Orthologous Groups
NR: Non-Redundant Protein Database databases
PHI: Pathogen Host Interactions
DFVF: Database of fungal virulence factors
CAZyme: Carbohydrate activity enzyme
CBM: Carbohydrate binding module
CYP450: Cytochrome P450
GH: Glycoside hydrolases
GT: Glycosyl transferase
PL: Polysaccharide Lyases
CE: Carbohydrate Esterases
AA: Auxiliary Activitie. PKS Polyketide synthase
NRPS: Non-ribosomal peptide synthase
SM: Secondary metabolite
T1PKS: Type I PKS
