# Supplementary material for: Genomic and transcriptomic survey of an endophytic fungus Calcarisporium arbuscula NRRL 3705 and potential overview of its secondary metabolites
Source: BMC Genomics. 2020 Jun 24;21:424. doi: 10.1186/s12864-020-06813-6 (PMC7315530; doi:10.1186/s12864-020-06813-6)
Supplement: Supplementary file 1 — Additional file 1. [file 12864_2020_6813_MOESM1_ESM.docx]

**Genomic and transcriptomic survey of an endophytic fungus *Calcarisporium arbuscula* and potential overview of its secondary metabolites**

**Jin-Tao Cheng^1,2^, Fei Cao****^1,2^, Xin-Ai Chen^1,2^, Yong-Quan Li^1,2^*, Xu-Ming Mao^1,2^***

**^1^Institute of Pharmaceutical Biotechnology, School of Medicine, Zhejiang University, Hangzhou 310058, China**

**^2^Zhejiang Provincial Key Laboratory for Microbial Biochemistry and Metabolic Engineering, Hangzhou 310058, China.**

***To whom correspondence should be addressed:**

**Prof. Xu-Ming Mao, E-mail: xmmao@zju.edu.cn; Tel: 86-571-88981335; Fax: 86-571-88208569.**

**Prof. Yong-Quan Li, E-mail: lyq@zju.edu.cn; Tel: 86-571-88206632; Fax: 86-571-88208569.**


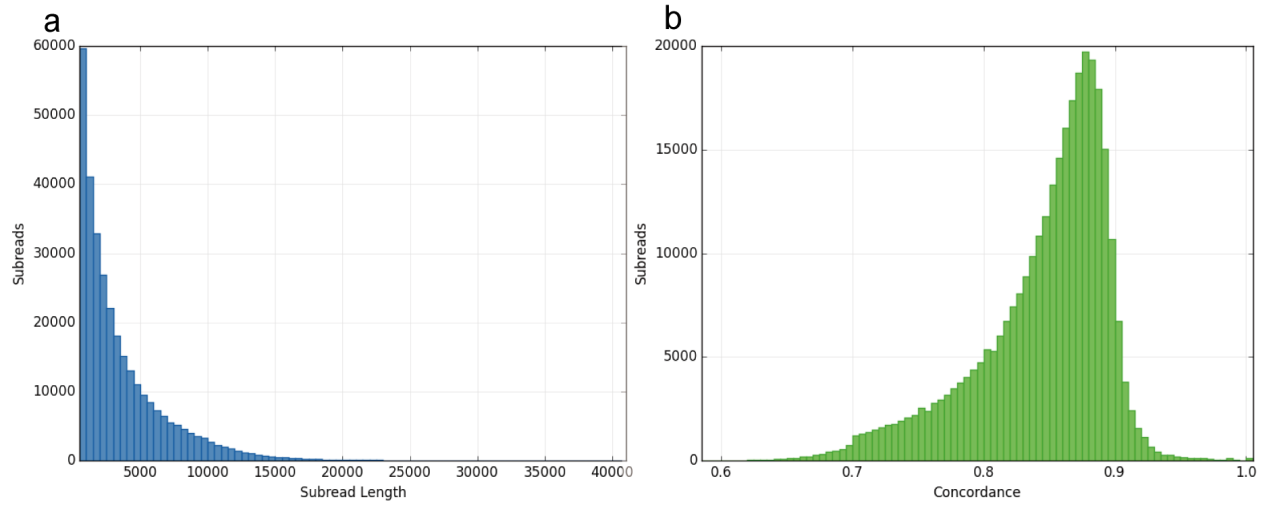
**Fig. S1 Statistics analysis for subread of *C. arbuscula* clean data**

**a** Sequencing read length of clean data. The left vertical and the right vertical represent the Subread length, number of Subread at specified length.

**b** Mass distribution of clean data. The abscissa indicates the quality of the sequencing, the column bar corresponds to the left ordinate, and the left ordinate indicates the number of reads corresponding to the sequencing quality.


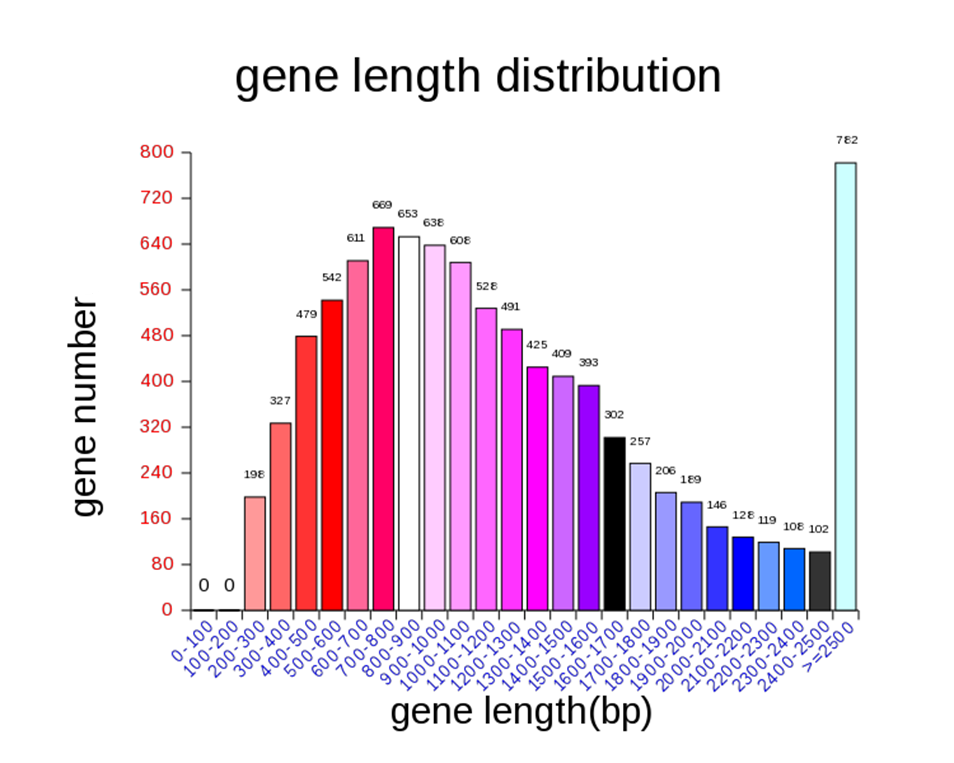


**Fig. S2 Statistics analys for gene length distribution of *C. arbuscula* clean data**


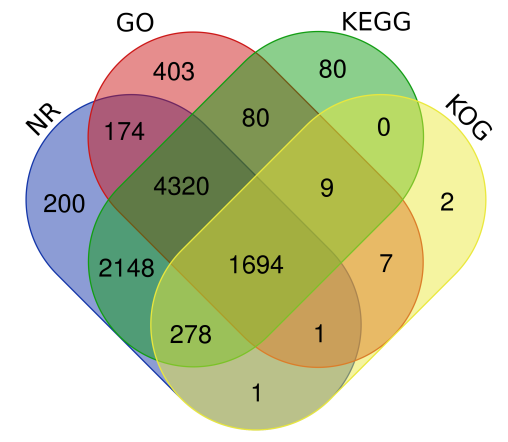


**Fig. S3 Venn-plot distribution of coding genes by four databases**


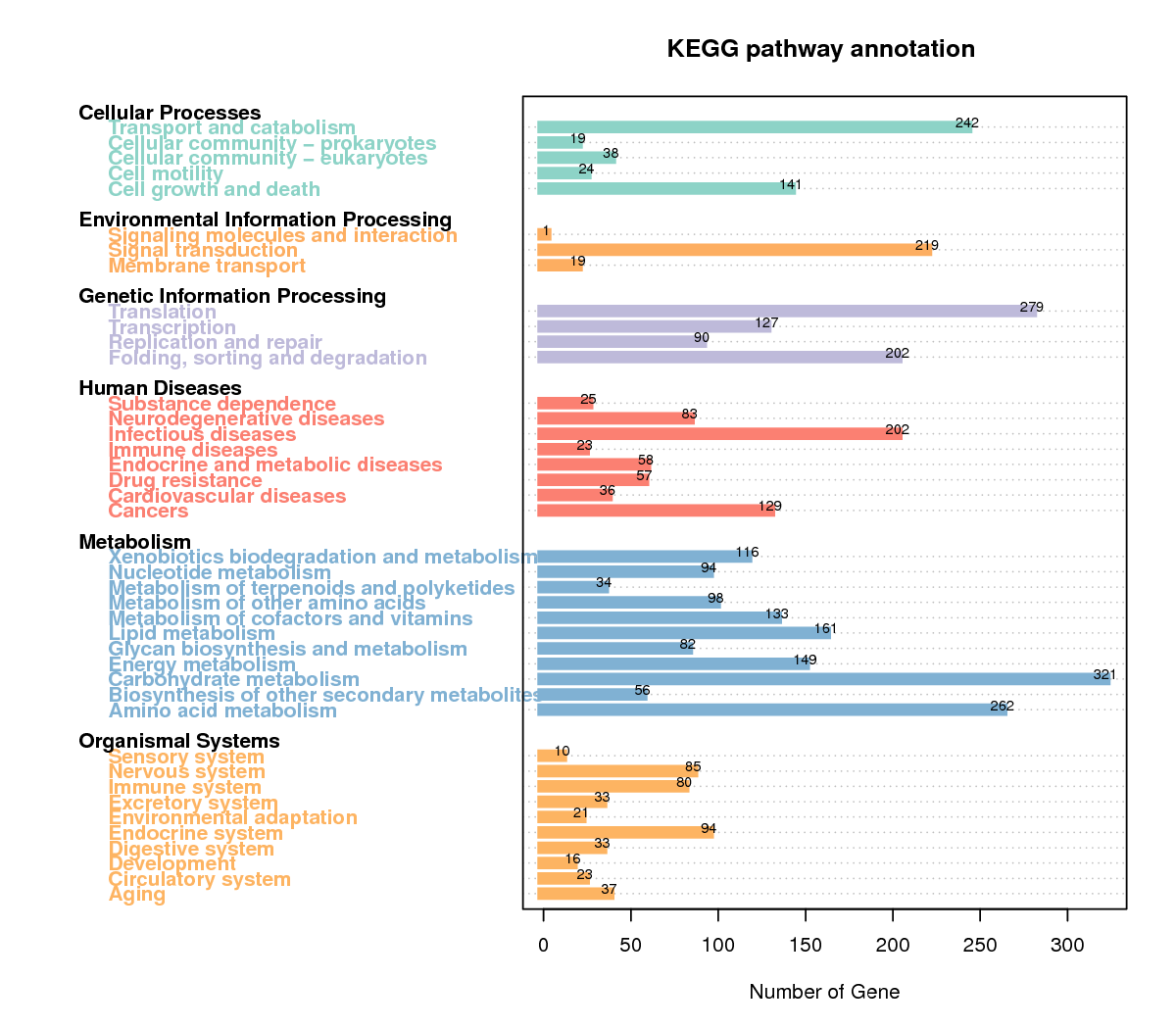


**Fig. S4 Histogram of KEGG distribution of predicted proteins of *C. arbuscula***


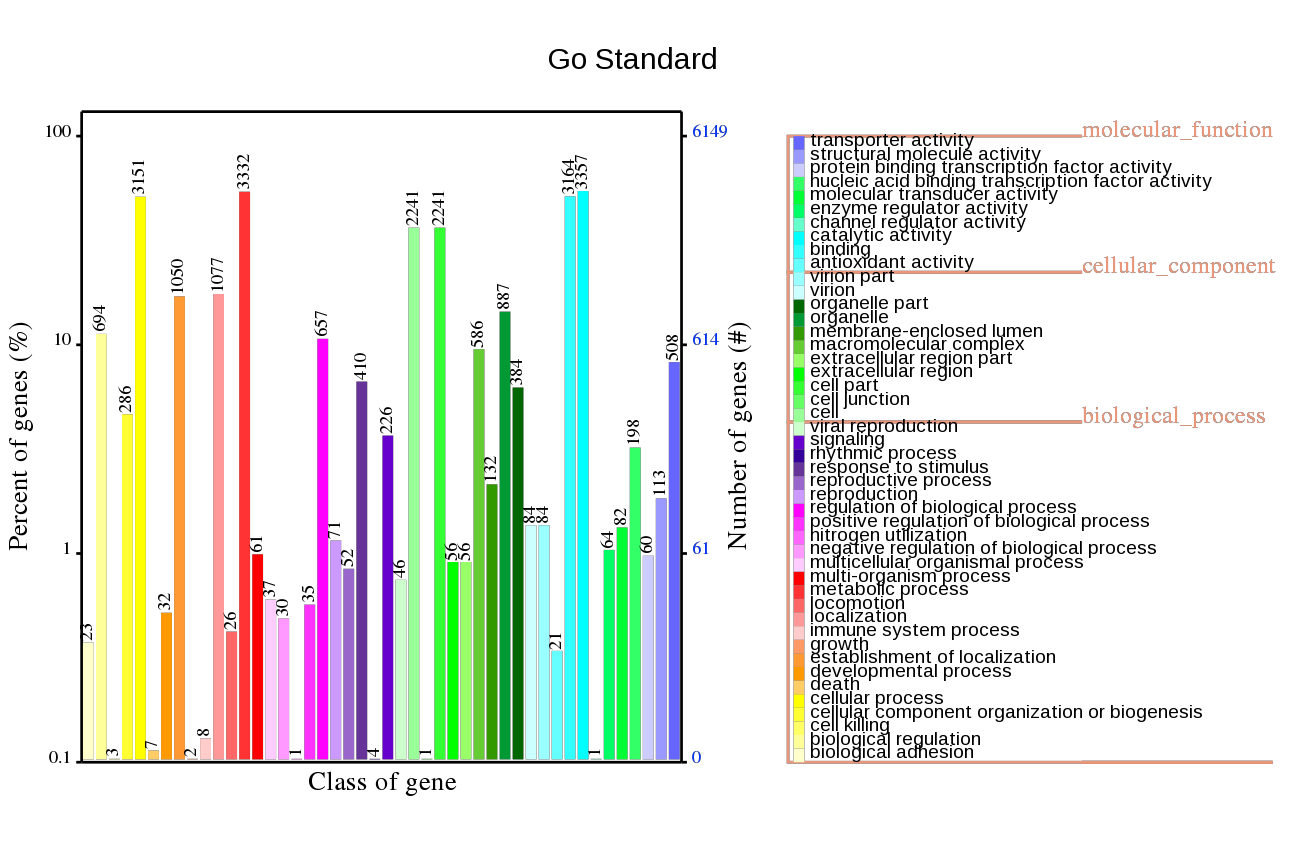
**Fig. S5 Histogram of GO distribution of predicted proteins of *C. arbuscula***


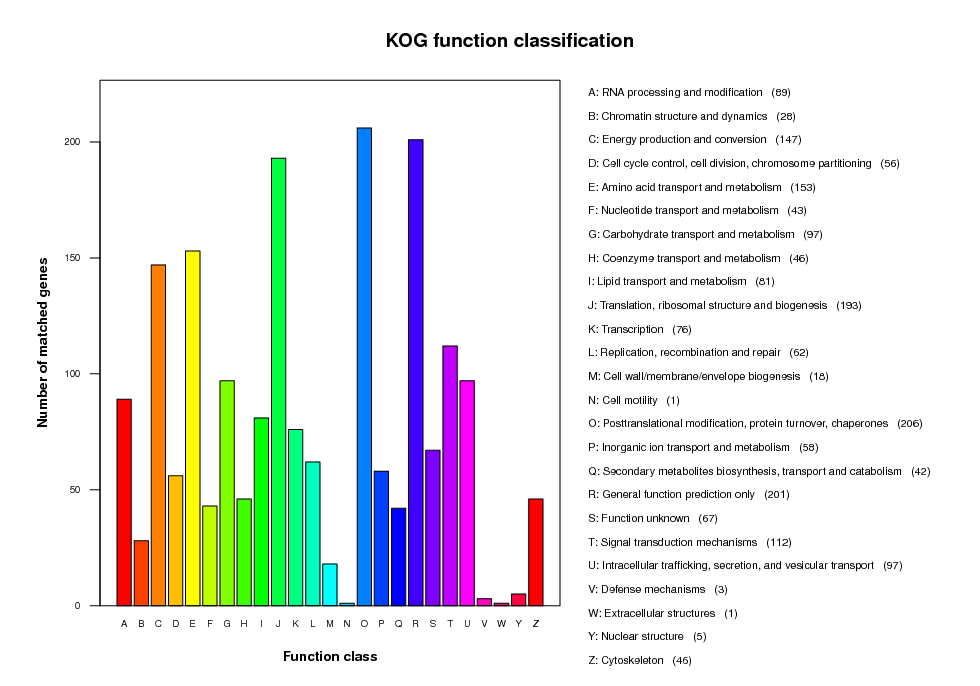


**Fig. S6 Histogram of KOG distribution of predicted proteins of *C. arbuscula***


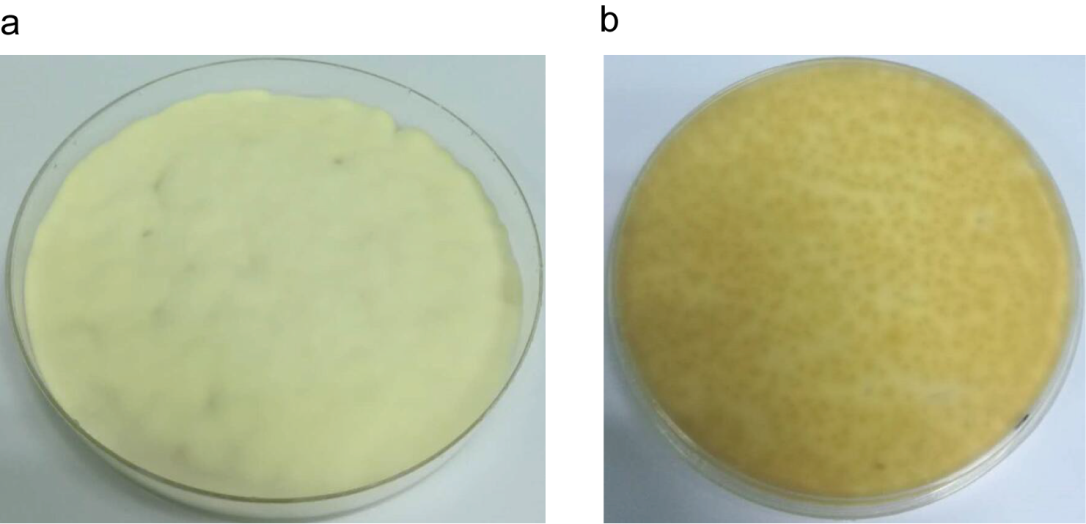


**Fig. S7 Sporulated mycelium of *C. arbuscula* on PDA plate after 5-day-culture at 25 °C**.

**a** Spore mycelium of *C. arbuscula* on PDA plate after 5-day-culture at 25 °C from the front of the plate. **b** Spore mycelium of *C. arbuscula* on PDA plate after 5-day-culture at 25 °C from the reverse side of the plate.


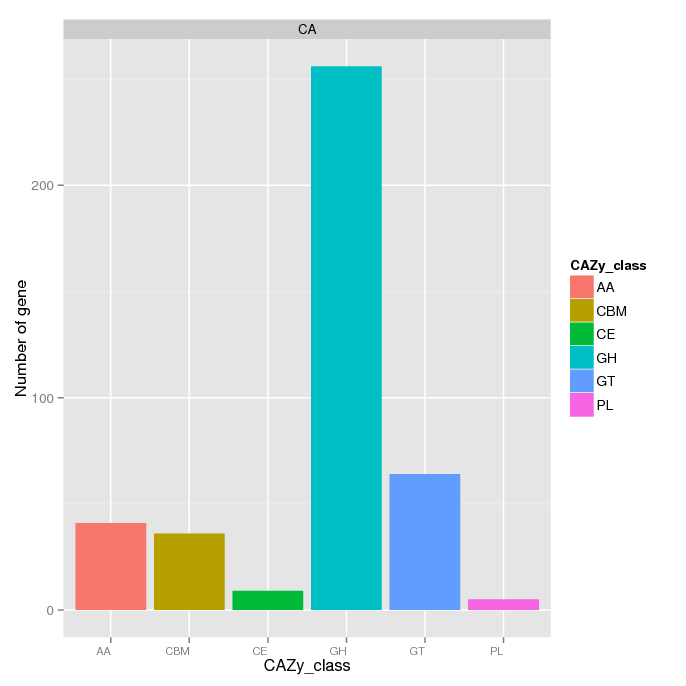


**Fig. S8 Histogram of CAZymes distribution of predicted proteins of *C. arbuscula***


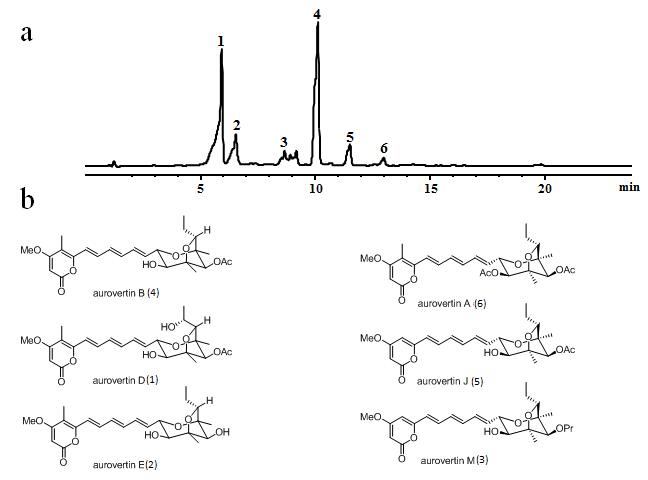


**Fig. S9 HPLC analysis of aurovertins from metabolites of *C.arbuscula.***


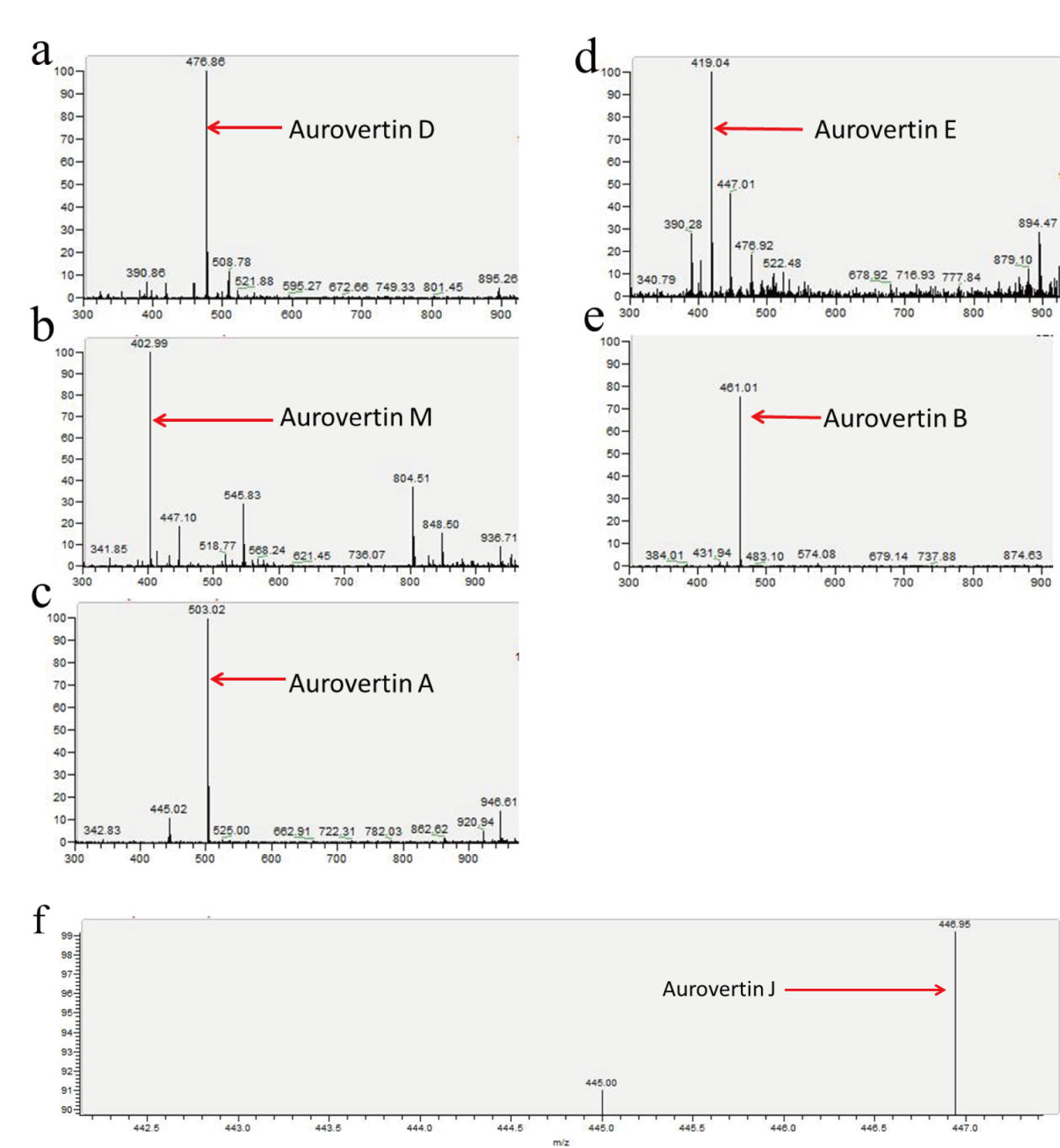


**Fig. S10 LC–MS analysis of aurovertins in positive ion mode from metabolites of *C.arbuscula.***

**
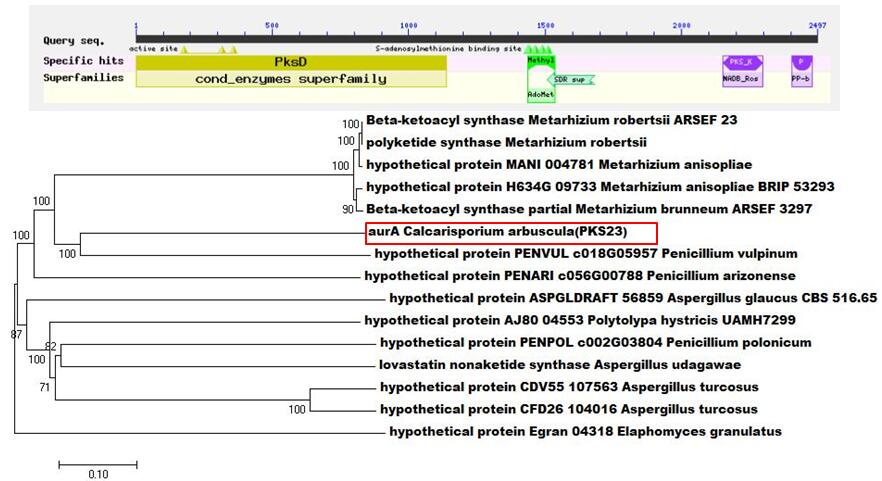
**

**Fig. S11. Conserved domain (CD-Search) and Distance tree results obtained fromNCBI BlastP analysis of predicted PKS of *C.arbuscula* NRRL3705 putatively involved in aurovertin synthesis (Neighbour-Joining, maximum distance >0.5; grisham mode) Comparison between conserved domains of *C.arbuscula* NRRL3705 SM Cluster 23 (PKS23) prediction and other known fungal genome assemblies– representation obtained with AntiSMASH software.**
